# Causality in digital medicine

**DOI:** 10.1038/s41467-021-25743-9

**Published:** 2021-09-15

## Abstract

Ben Glocker (an expert in machine learning for medical imaging, Imperial College London), Mirco Musolesi (a data science and digital health expert, University College London), Jonathan Richens (an expert in diagnostic machine learning models, Babylon Health) and Caroline Uhler (a computational biology expert, MIT) talked to Nature Communications about their research interests in causality inference and how this can provide a robust framework for digital medicine studies and their implementation, across different fields of application.

What is causality and how do causality and digital medicine interact in your field?

**Ben Glocker**: Causality is concerned with the modelling of the underlying cause-effect relationships in the data that we wish to analyze. Here, the language of causal reasoning allows us to formalize our knowledge about these relationships and any assumptions that we make regarding the so called data generating process which includes aspects of data acquisition, data collection, and data annotation. A detailed, causal description of the data generating process can be used to illustrate how the data has been generated and what factors influence the specific characteristics of a study sample. For example, using causal diagrams we can explicitly communicate what factors of variations affect the study population, the acquisition procedures, the annotation policies, or the inclusion/exclusion criteria. It is important to model and communicate the data generating process as this allows us to identify potential shortcomings, limitations and biases in our data which may impact the generalizability or even the validity of the conclusions we draw from statistical data analyses. In the field of machine learning for medical imaging, we often aim to build statistical models that take medical scans (and possibly other information) as inputs in order to make predictions about a patient’s disease status, the presence of pathology, or the effectiveness of treatment. Here, the underlying causal relationships between the inputs and outputs can have profound implications on the types of machine learning strategies we may want to employ. Further, we may be interested in identifying previously unknown causal relationships, for example, between imaging biomarkers and the efficacy of therapeutic interventions.

**Mirco Musolesi**: Providing a definition is very hard, since in my opinion, the concept of causality per se is deeply philosophical. I would define causality in very practical terms, also given my own work and background, and say that causality analysis allows us to answer cause-effect questions starting from real-world data. As far as digital medicine is concerned, causality analysis allows us to operationalise our analytical findings in a sense, because it literally enables us to use data to make informed choices. A possible example is the choice of the right therapies and interventions given the existing conditions and the external context. In fact, causal analysis allows us to understand the endogenous and exogenous factors that might have an impact, for instance, on a certain behaviour or medical outcome. It underpins our reasoning and it is of fundamental importance for evidence-based decision making. It is not sufficient to collect data, possibly in real-time and from a large population using digital technologies; interpreting the data from a causal point of view is essential to take informed action. The actual “feedback loop” might be implemented through the same digital technologies. This reasoning is true for situations involving individuals, but also for public health policies and interventions, like those that have been adopted by governments and local authorities during the current covid-19 pandemic.

I have been working in the area of real-time monitoring of physical and mental health using mobile sensing and through the collection of real-time data (e.g., from social media). I am interested in applying causal methods to this class of datasets also for understanding and planning effective feedback systems. Most of the existing work is based on correlation; as I said, deriving causality relationships from these datasets is fundamental for deriving actionable insights.

**Jonathan Richens:** Many of the routine questions that arise in clinical practice, such as “what treatment should I recommend?” or “why is the patient experiencing these symptoms?”, are fundamentally questions about cause and effect. Causality is a field of research that tells us how to answer these types of questions, and what assumptions and resources are required to do so. For example, one of the key tasks in digital healthcare is generating individualised care plans. This involves tailoring a sequence of decisions to a single patient, steering them towards the desired health outcomes, which in turn requires estimating the causal effect that each decision will ultimately have on the patient. Randomised control trials are the gold standard for establishing these cause-effect relations, but there are many situations where randomising these decisions would be unethical, unscalable or overly disruptive to the patient. So instead we typically have to work with observational data sets such as electronic health records, which only capture associations rather than bona fide causation. We use causal inference methods to bridge this gap and answer these causal questions, using observational data along with modelling assumptions. While this is the most studied application, causality has deep roots in clinical decision making that go beyond estimating treatment effects. For example, diagnostic reasoning involves generating and testing hypotheses for the most likely underlying cause of a patient’s symptoms. So even this textbook clinical decision problem is in fact a causal inference problem in disguise.

**Caroline Uhler**: Important questions in the biomedical sciences are inherently causal: which genes regulate one another? How does an intervention/perturbation (e.g. drug, over-expression, or knockout) affect the expression of all genes? And which intervention could move the system from a diseased state back to the normal state? Causal relationships between nodes, such as genes, can be represented by a directed network, where a directed edge from node 1 to node 2 means that node 1 directly regulates node 2 and thus perturbing node 1 changes the value of node 2. The biomedical sciences have genetic and chemical tools that allow perturbational screens on a scale that is unmatched by other fields. These features make the biomedical sciences uniquely suited to being not only one of the greatest beneficiaries of methods in causality, but also one of the greatest sources of inspiration for the field of causality.

What are the most important concerns related to non-causal approaches in your field, with respect to clinical and biomedical research?

**B.G**.: One of the most pressing challenges in the field of medical imaging is dataset shift, which concerns the issue of (unknown) changes between the development data used for building predictive models, and the real-world, clinical data we are facing after deployment. We know that dataset shift can have detrimental effects on the model performance. Here it is important to be able to identify the types of shifts, e.g., acquisition shift vs population shift vs annotation shift vs prevalence shift. Each type of shift requires different mitigation strategies. Some shifts are harder, some are more straightforward to tackle. Dataset shift can be well analyzed under the lens of causality as the types of shift are best described using a detailed, causal mapping of the data generating process. Dataset shift is also closely related to the issue of learning spurious correlations, which occurs when a signal or pattern in the input data is being picked up during model training that is predictive under the development data but non-predictive in the real-world test data after deployment. This can easily happen in imaging settings where, for example, site specific noise patterns manifest themselves in the raw data (caused by specific imaging hardware or image reconstruction algorithms). Purely associative approaches are susceptible to spurious correlations, as any signal that will help to establish a (non-causal) relationship between inputs and outputs may be learned as long as it helps to optimize the mathematical objective function. There is risk that this may lead to false discoveries such as the use of imaging to predict patient outcomes, while the true relationship may have been based on confounding factors.

**M.M**.: In terms of academic studies, the major risk I can see is related to the reporting of results of studies and their impact on policy and decision-making in general. Often unwillingly, these results are communicated to the public without distinguishing correlation from causation. Let us consider a typical example: the influence of green spaces on health on physical and mental wellbeing of people. You can run a correlational study without taking confounders into consideration. The problem is that the public and decision-makers might interpret these results in terms of causal effects, without considering the impact of important confounders, such as wealth and education levels in these areas. This is a simple example, but it applies to a variety of studies with the goal of improving the wellbeing and health of individuals.

**J.R.:** Non-causal approaches are totally appropriate for many tasks; they only become problematic when they are misapplied to tasks that fundamentally require causal inferences. A common issue is the use of non-causal methods for tasks that at some level involve predicting the outcome of interventions. One example of this is the widespread application of risk prediction algorithms to population health management, which assumes that patients with the highest risk will benefit the most from additional care. Clearly this is a strong assumption, and a more prudent approach is to assign resources to those who will benefit the most from them—i.e. using the causal “effect of treatment on the treated” rather than associative risk predictions. Another example is the use of user-generated data to validate and optimize digital health products. Confounding and selection biases greatly limit the validity of any insights derived from this data using non-causal methods. For example, if I passively observe that using a new digital health product correlates with a reduction in hospitalization rates, is the product preventing hospitalizations, or are younger people both more likely to use the product and less likely to be hospitalized? One source of optimism is that as digital technologies become more widely adopted, these causal inference problems become easier to solve. Digital health platforms can in principle capture all of the information exchanged between a patient and their care provider, placing strong limitations on unobserved confounding between treatment decisions and outcomes, and enabling robust causal inferences to a much greater degree than standard electronic health records.

**C.U**.: Unlike in early drivers of research in machine learning (ML) and artificial intelligence (AI) such as recommender systems or online advertising, in the biomedical sciences there are natural laws to be discovered, phenomena are physically interpretable, and predictive accuracy is often not sufficient, but causal mechanisms are the ultimate goal. Consider the following example: While in a recommender system it is sufficient if the model is able to predict that if someone buys sunscreen, then this person might also be interested in ice cream. High predictive accuracy is sufficient and it is not critical to understand the underlying causal mechanisms. On the other hand, consider a classical machine learning challenge in the biomedical sciences such as the ISBI-ISIC Melanoma Classification Challenge, where the goal is to distinguish benign from malignant skin lesions. We showed that in the 2017 dataset used for the challenge, neural networks trained to classify between benign and malignant lesions would recognize bandages in the benign lesions as features of malignant skin lesions. These neural networks were thus using non-causal features for the prediction. This would be highly concerning for its deployment in medical settings, thereby demonstrating the need for methods that can identify causal features in biomedical datasets.

Can you mention one or more examples of digital medicine studies where causal approaches have been (or would have been) applied successfully? Especially when compared to a simpler, associative framework.

**B.G**.: Recent work that we are particularly excited about is the development of new machine learning methodology for the generation of so called counterfactual images. There are a few groups working on the idea of incorporating causality into deep generative models in order to be able to synthesize realistic, subject-specific hypothetical images. For example, we can already reasonably well predict how a brain scan of a specific individual may look like if that person were 10 years younger or older. This requires approaches that go beyond association in order to predict the effect of particular interventions to the causal model relating an individual’s characteristics (e.g., their age, biological sex, etc.) to the anatomical manifestation of the brain visible in a medical scan. Such interventions require mathematical tools of causal reasoning, such as our recently proposed deep structural causal models. As a next step, we want to extend this work to study subject-specific effects of diseases, such as Alzheimer’s. Here, the generated counterfactual images could be used to answer questions like “how would this individual’s brain look like if they were healthy?”. This is different from today’s population-based analyses which mostly look for the average effect of a disease across the patient population. Another area where counterfactual images could be useful is to aide with the explainability of image-based machine learning methods. For example, the generated images could highlight anatomical changes that are deemed important for a specific prediction. Images are a natural and human-interpretable way of conveying information.

**M.M**.: I believe that one of the most interesting examples is the quantification of the effects of behavioural interventions. In terms of practical methods, I would say that researchers will benefit from analysis based on causality methods. Let us consider an example. Let us suppose that you want to design a study about strategies for improving physical and mental wellbeing using a mobile app. The app itself in terms of timing and content of notifications should be designed in such a way to allow for example random control trials. Analysing only the correlation between changes in behaviour without taking into consideration treatment and control groups according to a causal lens will be very limiting: it will not allow for quantifying the impact of different interventions, such as different types of messages sent in different contexts, time of the day, etc.

**J.R.:** One famous example where causal approaches could have been applied is a study from the University of Pittsburgh and Carnegie Mellon, which trained several machine learning algorithms to predict mortality risk and use this to triage pneumonia patients. Surprisingly, the algorithms learned to treat asthma as a protective factor against mortality—a dangerous conclusion that could result in less aggressive treatments, despite asthma in fact representing an increased mortality risk. This mistake was not due to algorithmic error; the training data really did appear to show a lower mortality risk for asthmatic patients due to a path-specific causal effect. Asthmatics had historically received more aggressive treatments than non-asthmatics, resulting in an overall reduction in mortality rate. Instead, this error derives from confusing a causal task, identifying the best intervention for a patient, with an associative task, predicting the patient’s risk. Recently, causal algorithms have been proposed that can avoid these pitfalls. For example, diagnostic algorithms have historically confused a causal task, identifying the most likely underlying cause of a patient’s symptoms, with an associative task, predicting the likelihood of a disease. As a result, these associative algorithms can make mistakes that violate common sense principles, like suggesting diseases that could not possibly cause a patient’s symptoms. On the other hand, causal diagnostic algorithms can avoid these mistakes and achieve significantly better performance.

**C.U**.: When the COVID-19 pandemic struck in early 2020, doctors and researchers rushed to find effective treatments. While drug discovery is still mostly done experimentally, computational methods are starting to be used and have already led to successful drugs. A popular approach is based on network analysis. An important resource for this is the protein-protein interaction database. By overlaying this network with information on the nodes of how differentially expressed a gene is in the diseased state as compared to the normal state, nodes can be found that are either very central to, or connected to, many nodes that are differentially expressed by the disease. These central nodes in the disease interactome are candidates for drug targets.

A protein-protein interaction network is an undirected network that does not capture any regulatory or causal relationships. While popular network-based approaches screen for drugs that target nodes that are connected to many nodes that are affected by a disease, if these target nodes are downstream of nodes affected by a disease, the drug will have no effect on the disease nodes. It is thus critical to infer a causal network to identify which genes and proteins are “upstream” (i.e. they have cascading effects on the expression of other genes) and which are “downstream” (i.e. their expression is altered by prior changes in the network). An ideal drug candidate would target the genes upstream of the genes that are differentially expressed by the disease. By using causal structure discovery algorithms to turn the undirected protein-protein interaction network into a directed network, in recent work we identified the protein RIPK1 as a promising target for COVID-19 drugs. Interestingly, it has been shown that RIPK1 directly binds to SARS-CoV-2 proteins, and a RIPK1 inhibitor is currently in clinical trials for COVID-19.

What are the biggest obstacles preventing wider application of causality approaches, from your perspective?

**B.G**.: Applying causal reasoning requires certain assumptions to be made about the underlying relationships in our data, and many of those assumptions will remain untestable. A key criticism of causal approaches relates to the issue of unknown confounding, meaning we often cannot be certain that there are no other factors that explain the effects we see in our (observational) data. Making appropriate assumptions requires domain expertise, so the field of causal reasoning naturally requires a multi-disciplinary approach.

**M.M**.: One of the major issues is related to the way education and training in quantitative and statistical methods are currently structured. Causal methods are not routinely taught in secondary schools and University courses, except perhaps in core Statistics, Epidemiology, and possibly in certain areas of Social Sciences (Economics and Political Science come to mind), at least at Undergraduate level. There are a variety of factors that are associated with the current landscapes, including the fact that the number of modules allocated to statistical methods is usually quite limited - often students take only 1 Statistics module in their degree. Non-causal approaches are not trivial per se for students not trained in statistical methods. I believe that the situation might be improved if Statistics and Data Science training could start earlier, ideally in secondary school.

Causal inference methods are also not mainstream in certain communities in Computer Science either and, for this reason, it is not common to see applications of these techniques outside Machine Learning and related disciplines. I personally experienced situations where the reviewers of my papers in which we applied causal reasoning did not appear completely comfortable with the experimental setting and analysis so that it was difficult for them to champion the paper for publication.

Other obstacles are related to the fact that, in order to be able to perform a causal analysis of the data, it is necessary to collect information about a potentially large population and for a long period of time. This is often practically hard, for example when the data are collected using mobile applications. In fact, it might be difficult to ensure compliance by keeping a large number of users involved in a study for a considerable amount of time.

**J.R.:** While machine learning can be a powerful tool in medicine, it can also be a procrustean bed. There is an understandable desire to apply the latest machine learning methods to medical problems, but most of these methods are limited to making associative inferences. This tendency to try and fit the problem to the tool has been an issue throughout the history of AI in medicine, and while the classic medical textbooks clearly emphasize the importance of causal reasoning, this has often been ignored in the AI community. However, this state of affairs is rapidly changing. Recent years have seen the application of more advanced regression and representation learning techniques to causal effect estimation, which has done much to bridge this gap between causality and modern machine learning methods. Still, these causal methods require additional domain knowledge and validation compared to non-causal methods, which acts as a further barrier to entry. Likewise, a lack of knowledge of causality has limited the uptake of causal methods in applied research. But this is also rapidly changing, with both large and medium size tech companies now investing heavily in causal research.

**C.U**.: The main difficulty in biomedical applications is that one cannot assume that the underlying causal network is known. The causal mechanisms are often exactly what researchers are after. It is well-known that from observational data alone, i.e. data collected in a passive fashion by observing a system rather than acting on it, one can only partially identify the underlying causal network, and even this only under strong assumptions. A full understanding of the causal relationships in a system requires interventional data that is obtained by deliberately and carefully altering one or more components of the system.

Unlike most other disciplines, the biomedical sciences have chemical (e.g. drugs) as well as genetic (e.g. knock-out, knock-down, over-expression) tools to deliberately alter components of the system, as well as technologies such as single-cell RNA-seq or imaging to obtain high-throughput observational and interventional data. This has resulted in large-scale perturbational datasets including CMAP, DepMap and Cell Painting with sequencing or imaging readouts of single drug or gene perturbations. While perturb-seq and optical pooled screens allow for combinatorial perturbations (i.e. the perturbation of multiple genes at once), large-scale combinatorial perturbation screens are not yet readily available and would be critical for motivating new causal methods as well as validating current causal structure discovery methods.

Together with new datasets, the field also needs a theoretical and algorithmic framework for causal inference based on interventional data. In particular, even if large-scale combinatorial perturbation screens become readily available, it will be infeasible to perform all combinations of perturbations. Data acquisition and causal inference methods thus will have to go hand-in-hand in order to identify the most informative perturbations, ideally in an iterative fashion using an active learning approach. In addition, the ability to perform large-scale interventions not only provides a toolkit for *learning* the causal structures underlying biological phenomena, it also provides a strategy for *controlling* them. While control theory and causality have had few interactions thus far, when taking an intervention-centric approach to causality, it is natural to consider control theory given its focus on identifying the best actions to control a system or move it towards a desired state.

Together with Anthony Philippakis I co-direct the newly-launched Eric and Wendy Schmidt Center (EWSC) at the Broad Institute, which aims at understanding the programs of life. Recognizing the importance of causal inference for such an undertaking, causality is a central theme of the center. A key goal of the EWSC is to not only bring the tools of modern ML into the biomedical sciences, but to also make the biomedical sciences a key driver of foundational advances in ML. Importantly, this includes identifying and generating key biological datasets that are critical for advancing developments in causal inference that can then feedback to biological discovery.

Can you give us a quick walkthrough on how causal approaches can be implemented in a digital medicine study in your field, starting from data collection?

**B.G**.: Assuming the goal is to build a predictive model, a first question to ask could be about the causal relationship of the input data and target predictions. What do we know or what do we assume about the causes and effects in our data. We could then try to map this out in a causal diagram which could be extended to include aspects of the data collection such as quality control, selection criteria, labelling, and any other factors that may impact the variation of the data. All this may be done even before any actual data has been collected, as it may inform our planning (e.g., is it sufficient to collect data from a single site, is our study population representative, etc.). The causal relationships in the data may further dictate what types of machine learning methodology we may want to employ. Some learning strategies, such as semi-supervised learning, may be non-optimal in certain settings. So even if we opt for purely associative approaches for the data analysis, such as deep convolutional neural networks, a causal pre-analysis of our study setting may be helpful to avoid common pitfalls in machine learning.

**M.M**.: As I said, I usually work with datasets that have been collected through mobile applications or extracted using APIs. When you design (mobile) applications for data collection you tend to collect as much data as possible in order to consider all possible confounders. In this case, as researchers we have control of the data collection process and, therefore, a well-designed study allows us to apply causal analysis techniques with very few limitations. However, the practice might be rather different from the theory. In fact, it is often the case that it is not possible to collect all the required information for an “ideal study”, usually for privacy reasons. Let us consider for example the mobile applications used for alerting users for potential exposure to covid-19, i.e., the so-called exposure notification apps. In theory, these apps could have been designed to collect many variables of interest for understanding the spreading of the virus in populations, such as, for example, the network of contacts of infected individuals, their timing and the location where they took place, etc. In certain countries, this is the case, but causal analysis is not possible with apps based for example on the Apple/Google Exposure Notification API (actually, given the API limitations by design, correlation analysis is also hard). This is an extreme case, but in many situations, causal analysis is not possible since key variables cannot be monitored because of legal and/or ethical considerations.

Another interesting aspect is related to the selection of the participants. In fact, depending on the way a mobile application is distributed, researchers might not have full control on the selection of participants. A typical example is the case of applications that are advertised broadly and distributed through app markets, such as the Apple App Store and Google Play Store. In these cases, experimenters do not have direct control on the selection of the participants. In theory, it would be necessary to control for variables such as demographics, but this is not always possible, because this information might not be available. For example, if the ethical review of a study does not allow for the collection of demographic information, this cannot be inferred through the app stores, since they do not record this type of information.

Another typical situation is the case of causal inference applied to “big data(sets)”, such as those extracted by means of social media APIs. In these situations, again, the actual composition of the set of participants” is not directly available, since they are a sort of “exhaust”, as some people say, which comes from the normal use of the platform, i.e., they are secondary data. In these situations, caution in drawing conclusions in terms of causality (but also in terms of correlation) is essential. At the same time, luckily enough, we have a plethora of powerful techniques for dealing with these datasets and extracting causal relationships if present.

**J.R.:** Causal diagnostic algorithms use explicit causal models to make inferences, and the first and most challenging step is learning a pathophysiological model. These are typically large graphical models representing symptoms, diseases and risk factors, situated in a directed acyclic graph (DAG) which encodes their causal relations. The DAG is constructed using a knowledge graph derived from medical literature and expert knowledge, or it can be learned from health records and epidemiological data using causal discovery algorithms. The model parameters are then learned using a combination of expert elicitation, electronic health records and epidemiological data, typically making assumptions of parameter modularity and independence to expedite learning and improve interpretability. Diagnosis is a dynamic and investigative process involving many sub-tasks such as evidence gathering, hypothesis generation, and deductive reasoning. So the next step is to devise inference algorithms for each of these tasks. For example, associative inference can be used for evidence gathering, while causal inference can be used to weight diagnostic hypotheses. Finally, the complete system has to be validated and extensively tested for accuracy and safety.

**C.U**.: My group has worked extensively on the development of a theoretical and algorithmic framework for causal structure discovery, i.e. the identification of causal relationships, from a mix of observational and perturbational data. An example of how such methods can be applied to pinpoint candidate drug targets has been discussed above in the context of COVID-19.

Given the urgency in the COVID-19 pandemic, it is of particular interest to identify drugs that are already FDA approved and are also effective against COVID-19 (this problem is known as drug repurposing). Given large-scale drug screens performed for other diseases (such as CMAP described above, which concentrates on cancer cell lines), the problem then becomes to transport the effect of a drug from one disease context to another and predict the effect of drugs on SARS-CoV-2 infected cells knowing their effect on cancer cell lines. This problem is known as causal transportability or synthetic controls/interventions in the causality and policy evaluation literature. Alternatively, this problem can be seen as a style transfer problem in machine learning, where the style is the effect of a drug and the goal is to transfer it from one disease context to another. Autoencoders and generative adversarial networks have been very successfully applied for style transfer in computer vision applications by viewing the style as a vector in the latent space that can be appended to an image to change its style. Such an approach for drug imputation can only work if the effect of a drug is aligned in the latent space across disease contexts. We showed in recent work that over-parameterized autoencoders, i.e. autoencoders where the latent space dimension is larger than the input dimension, can be used to enhance alignment and transport the effect of a drug from one disease context to another. This approach may be used to efficiently screen a large number of drugs virtually and then perform experiments only on a subset of candidate drugs in the new disease context.

New guidelines for AI-based clinical trials have been published recently. How do you think causality should fit in there?

**B.G**.: Causal reasoning requires us to be explicit about our assumptions. For example, we can present the assumed data generating process in form of a causal diagram which is intuitive and easy to interpret. This also means that our assumptions are made more transparent and can be scrutinized by others. In this way, the toolbox of causality can support better reporting of AI-based studies and help with the identification of potential issues such as dataset shift, sample selection and other biases.

**M.M**.: As a computer scientist, first, I am always careful in using the word Artificial Intelligence (AI). For me, AI has a specific meaning, which is related to the efforts in building artificial systems that can complete tasks that require human intelligence. For this reason, I would prefer the term ML-based clinical trials instead.

In general, I would welcome the adoption of the recent developments in causal inference, in particular those introduced by the machine learning community in the past decade. I would say that the adoption is slow, but it is definitely happening. Another fundamental aspect in this context is the interpretability of the algorithms used for designing the trials and analysing the outcomes. This is an essential area of research also for increasing the confidence of researchers and practitioners in these areas. I also personally see a need for tools for guiding causal reasoning and supporting reproducibility of the studies based on the results of these trials.

**J.R.:** The SPIRIT-AI and CONSORT-AI guidelines extend existing guidelines for randomised control trials to deal with AI based interventions. Similar guidelines also exist for the design and reporting of observational causal inference studies, and extending these seems like a natural next step given the growing importance of causal inference in developing and monitoring AI based digital health products. For example, many causal inference studies involve counterfactual queries, and there are no counterfactual data points that can be used to validate the accuracy of these predictions. So it is important to ensure high-quality reporting on the modelling assumptions used to make these causal inferences, as well as reporting sensitivity analyses, which are used to determine the potential effect of unobserved confounding on these studies.

**C.U**.: Exciting times lie ahead for the use of AI-based methods in clinical trials. Causality is an important tool in every aspect of drug development and clinical trials, from the identification of targets (see above), to toxicity estimation and dose estimation (for example using causal structure discovery or causal transport methods discussed above), to the selection of patients for the trial. As discussed above, machine learning based algorithms in clinical applications can cause harm, in particular when they are trained on datasets that are not representative of the one they are applied to. An important concern for the use of AI-based methods in clinical trials is that they may perpetuate and exacerbate underlying biases and health disparities. In particular, algorithms which at first glance seem agnostic to group membership, may exhibit disparate impact. Causality has recently emerged as a powerful tool to investigate such questions of algorithmic fairness. In particular, counterfactual reasoning can be used to investigate whether the outcome would have been the same for an individual if (s)he would have belonged to a marginalized group. Thus, causality could become an important tool to investigate fairness in clinical trials, select the right patients for the trial, and thereby help lessen health disparities.

**Figure:**
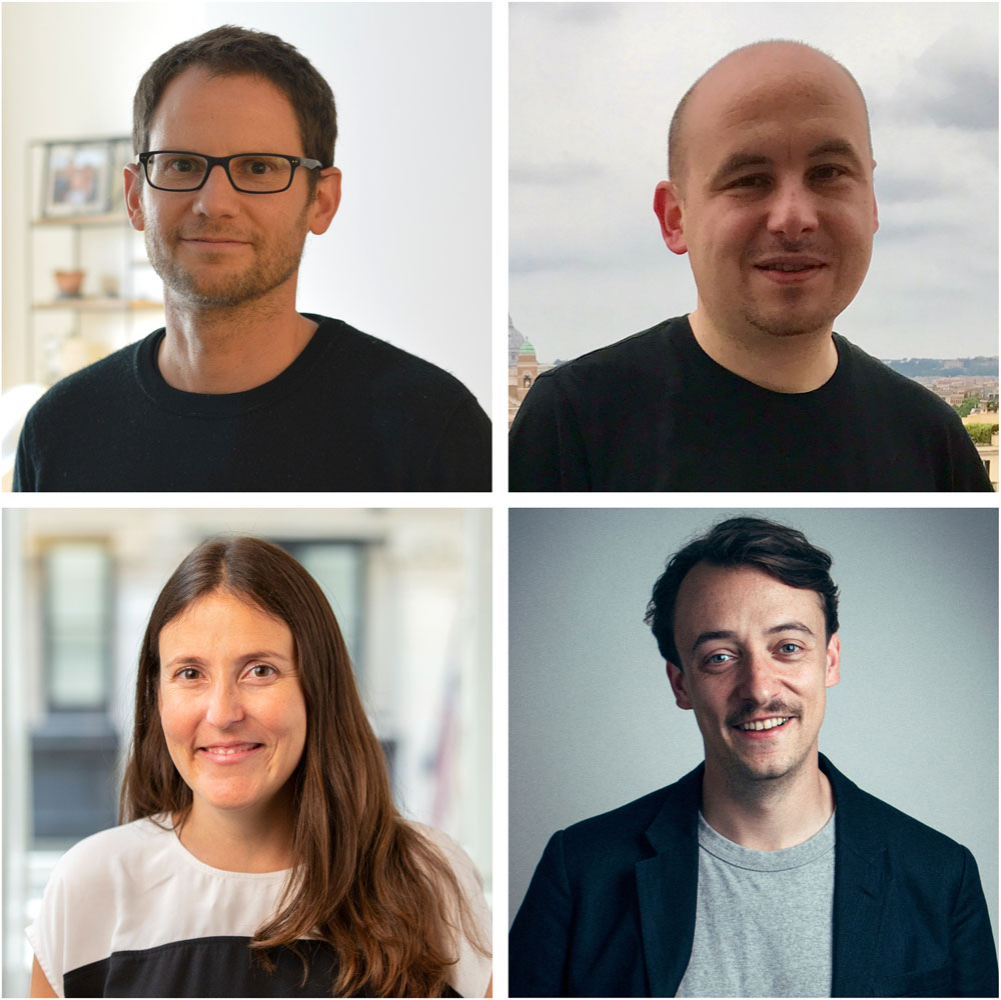
Ben Glocker, Mirco Musolesi, Jonathan Richens, Caroline Uhler

